# Economic development and road traffic injuries and fatalities in Thailand: an application of spatial panel data analysis, 2012–2016

**DOI:** 10.1186/s12889-019-7809-7

**Published:** 2019-11-04

**Authors:** Rapeepong Suphanchaimat, Vorasith Sornsrivichai, Supon Limwattananon, Panithee Thammawijaya

**Affiliations:** 1 Division of Epidemiology, Department of Disease Control, Ministry of Public Health, Nonthaburi, Thailand; 2 International Health Policy Program (IHPP), Ministry of Public Health, Nonthaburi, Thailand; 3 Health System Management Institute, Prince of Songkla University, Hat Yai, Thailand; 4 Faculty of Pharmaceutical Sciences, Khon Kaen University, Khon Kaen, Thailand; 5 Division of Innovation and Research, Department of Disease Control, Ministry of Public Health, Nonthaburi, Thailand

**Keywords:** Traffic accident, Traffic injury, Traffic fatality, Traffic death, Case fatality rate, Negative binomial regression, Random effects, Spatial data, Gross domestic product

## Abstract

**Background:**

Road traffic injuries (RTIs) have been one of the most critical public health problems in Thailand for decades. The objective of this study was to examine to what extent provincial economy was associated with RTIs, road traffic deaths and case fatality rate in Thailand.

**Methods:**

A secondary data analysis on time-series data was applied. The unit of analysis was a panel of 77 provinces during 2012–2016. Data were obtained from relevant public authorities, including the Ministry of Public Health. Descriptive statistics and econometric models, using negative binomial (NB) regression, negative binomial regression with random-effects (RE) model, and spatial Durbin model (SDM) were employed. The main predictor variable was gross domestic product (GDP) per capita and the outcome variables were incidence proportion of RTIs, traffic deaths and case fatality rate. The analysis was adjusted for key covariates.

**Results:**

The incidence proportion of RTIs rose from 449.0 to 524.9 cases per 100,000 population from 2012 till 2016, whereas the incidence of traffic fatalities fluctuated between 29.7 and 33.2 deaths per 100,000 population. Case fatality rate steadily stood at 0.06–0.07 deaths per victim. RTIs and traffic deaths appeared to be positively correlated with provincial economy in the NB regression and the RE model. In the SDM, a log-Baht increase in GDP per capita (equivalent to a growth of GDP per capita by about 2.7 times) enlarged the incidence proportion of injuries and deaths by about a quarter (23.8–30.7%) with statistical significance. No statistical significance was found in case fatality rate by the SDM. The SDM also presented the best model fitness relative to other models.

**Conclusion:**

The incidence proportion of traffic injuries and deaths appeared to rise alongside provincial prosperity. This means that RTIs-preventive measures should be more intensified in economically well-off areas. Furthermore, entrepreneurs and business sectors that gain economic benefit in a particular province should share responsibility in RTIs prevention in the area where their businesses are running. Further studies that explore others determinants of road safety, such as patterns of vehicles used, attitudes and knowledge of motorists, investment in safety measures, and compliance with traffic laws, are recommended.

## Background

Road traffic injuries (RTIs) are amongst the most critical public health problems in many countries. It is estimated that RTIs are currently the eight leading cause of death globally [1]. The World Health Organization (WHO) suggested that in 2010 over 1.24 million deaths were attributed to traffic accidents, and this figure now rose to 1.35 million in 2018 [1, 2]. Should the trend of RTIs continue at the current pace, traffic deaths will become the fifth leading cause of death worldwide by 2030 [1].

Traffic fatalities tended to be concentrated amongst the youth and young adults aged between 15 and 29 years, rendering a massive loss of the most productive years of human capitals [3]. Causes of RTIs can originate from various factors: poor driving skill, alcohol consumption, violation of traffic laws, poor vehicle conditions and unsafe traffic environments—to name a few. Thomas et al. suggested that human cause accounted for over than half of all causes combined [4].

Not only health loss, but economic consequences of RTIs are truly troublesome since RTIs led to a loss of a country’s gross domestic product (GDP) by about 3% on average [2]. Note that the economic damage from RTIs was more pronounced in low- and middle-income countries (LMICs) relative to high-income countries (HICs) [2].

In recent years, there have been many global initiatives to curb RTIs crisis. One of the most remarkable initiatives is the United Nations General Assembly Resolution 64/255, which proclaimed a ‘Decade of Action for Road Safety’ (2011–2020) [5]. The Resolution urged all stakeholders, including policy makers, academics, civil groups and international development partners, to provide synchronised effort to topple down RTIs catastrophe.

Thailand is an upper-middle income country where RTIs crisis has been one of the most serious public health concerns. The fast economic growth and its geographical location that connects the India Ocean with the Pacific Ocean make the country become the centre of logistics and transportation in the Indochinese Peninsula. These factors, inter alia, contributed to the vast volume of RTIs. According to the WHO survey amongst 180 countries in 2015, Thailand became the second deadliest country for road accidents, behind Libya [1, 6]. A recent report conducted by the Thailand Development Research Institute in 2017 suggested that RTIs led to a 6%-loss of the country’s gross domestic product (GDP) or 545 million Baht (US$ 16.5 million, based on the exchange rate of 33 Baht per US$ 1) [7]. The total loss of disability-adjusted life years due to RTIs was about 673,000 [8]. Male victims outnumbered females by approximately threefold and about four-fifth of the fatal victims were motorcyclists [8]. Of the affected riders, one third had history of alcohol use before the ride [9]. The enforcement of helmet law was quite low. The proportion of riders with helmet use was only 43.7% nationwide [10].

To address this cataclysmic public health crisis, the Thai Government has launched a number of RTIs-preventive strategies, including the establishment of the National Road Safety Management Centre chaired by Deputy Prime Minister and the foundation of Country Cooperation Strategy (CCS) on Road Safety, 2017–2021. The CCS serves as a mechanism for the Thai Government, the WHO, and numerous domestic partners to fuel investment in RTIs-related research and policy advocacy [11]. So far, numerous traffic prevention policies have been in place; for example, motorcycle lane enforcement, extensive campaigns for safe transportation during public holidays, amplifying the punishment on drunk drivers in the revised traffic laws, and banning alcohol advertisements on national television and radio programmes [12].

Yet, at the subnational level, the success of RTIs prevention varied hugely. It is found that, in 2012 the top-five provinces with largest RTIs toll consisted of Bangkok, Samut Prakan, Phranakorn Sri Ayutthaya, Chiang Mai, and Chiang Rai [13]. On the face of it, these provinces are areas with high economic activities. Also, the Thailand National Status Report on Road Safety reported that the central region—the most economically well-off region—encountered the largest gross traffic deaths per population relative to other parts of the country [13]. This impression contradicted the WHO report on global RTIs situation, which suggested a reverse relationship between RTIs and macro-economy [1].

While there are a great deal of research on risk factors of RTIs, studies that delve into economic implication on RTIs are quite sparse. Besides, most of the studies focusing on the relationship between RTIs and macro-economy were performed in developed nations, such as He et al. from Russia [14], Kulović and Dogdibegović-Kovač from Bosnia and Herzegovina [15] and Anbarci et al. from the US [16], while those in LMICs are still lacking.

Therefore the main objective of this study is to identify the association between RTIs incidence and provincial economic level in Thailand. It is hoped that a better understanding on this issue can help fill gaps in knowledge on the interface between RTIs and macro-economy; and it will also elucidate whether and to what extent the RTIs situation in HICs is similar to LMICs by using Thailand as a case study. Moreover, policy makers and health practitioners may utilise the findings from this research as a guide for prioritising the intensity of RTIs prevention in provinces with varying economic status. We also expect that the application of various statistical techniques (detailed later in Methods section) can academically add some values on the state of the art in the areas of econometrics and public health epidemiology.

## Methods

### Study design

We applied quantitative secondary data analysis on time-series data. This study can be viewed as an ecological study where the unit of analysis was a panel of 77 provinces in a six-year time span (2011–2016), resulting in 385 observations in total.

### Data sources and variables

The main interested outcomes were (i) traffic injuries (including deaths, minor injuries and morbidities), (ii) traffic fatalities (deaths) and (iii) case fatality rate (deaths per victim). These data were obtained from diverse sources. We explored the data on traffic injuries from the E-claim dataset, which compiled all traffic accidents reported to the Road Accident Victims Protection Company (RVP). The RVP is a private enterprise under state’s control, serving as the main national platform for all claim petitions and compensations related to RTIs. It should be noted that, aside from the E-claim dataset, there are other alternative sources, for instance, the Royal Thai Police Information System (POLIS) and the Injury Surveillance System (IS) of the Ministry of Public Health (MOPH). Yet, after exploring such alternative sources, we opted to select the E-claim dataset. This is because the E-claim contains the largest tolls of injuries.

Concerning traffic deaths, we relied on the combined dataset of the Division of Non-Communicable Diseases of the Department of Disease Control (NCD-DDC), the MOPH. The NCD-DDC’s website is now considered the most complete publicly-accessed data source for traffic fatalities [17]. It combined information from the E-claim, the POLIS, and the Death Registry together and checked for duplicates.

We then divided the total traffic injuries and traffic deaths by provincial population volume to obtain incidence proportion, and constructed case fatality rate by dividing traffic deaths by all injury cases. The size of population in a province was acquired from the Office of the National Economic and Social Development Board (NESDB).

The covariates of concern were region (Bangkok and its vicinity [so-called, Greater Bangkok], central, northeastern, northern, and southern regions), GDP per capita (in Baht), contribution of accommodation and restaurant businesses (as % GDP), contribution of manufacturing and industry businesses (as % GDP) and amount of gasoline purchased (in litres per capita). We explored two additional data sources to obtain these variables: (i) the NESDB, and (ii) the Department of Energy Business (DEB), the Ministry of Energy. GDP per capita was retrieved from the NESDB. We assumed provincial GDP per capita as a proxy for provincial economic level. Percentages of economic contribution by accommodation and restaurant businesses and by manufacturing and industry businesses were obtained from the NESDB. We used these variables as a proxy for urbanisation since in urbanised cities the rise of touristy and industry businesses might provoke hasty riding/driving behaviours amongst the middle-age population. The amount of gasoline sold at gas stations in each province in a year was retrieved from the DEB. We used this variable as a substitute for traffic intensity instead of the number of vehicles specified as counted by the name of provinces inscribed on the license plate. The use of gasoline volume provided us some advantages over the use of vehicle quantity due to the following reasons. First, gasoline amount better reflected the actual utilisation of vehicles in a particular area. Second, the name of provinces issued on the license plate was basically based on the vehicle purchasing sites rather than the real residence of the riders/drivers. Thus economically well-off provinces with large motor retails were likely to face exaggerating number of vehicles and this would cause a risk of misclassification bias in the analysis.

### Data analysis

All statistical analyses were performed by Stata v14.0 (StataCorp LP, College Station, Texas, US—serial number: 401406358220). We divided the analysis into two parts: (i) descriptive statistics and (ii) econometric analysis.

In the first part, cumulative incidence of RTIs over the whole country was calculated. Results were displayed by mean and median. All predictor variables were analysed in the same fashion.

In the second part, we started with data visualisation by scatter plots. Incidence proportion (for both traffic injuries and deaths) and case fatality rate were plotted against GDP per capita in natural log scale. The regression slope of each scatter plot was calculated. Subsequently, we had applied certain econometric techniques, namely, negative binomial (NB) regression and spatial panel data analysis. We opted to use the NB regression as the data pattern followed Poisson distribution with over-dispersion. In this stage, we further split the analysis into two sub-models: first, (conventional) (NB) regression, and second, NB regression with random effects, so-called, the RE model. We did not use fixed-effects (FE) model because, mathematically, the time-invariant variable (in this case, region) would have been cancelled out.

In spatial panel data analysis, we hypothesised that both independent and dependent variables were spatially correlated—for instance, provinces with relatively similar degree of incidence and/or similar degree of prosperity might be clustered together. In this respect, the spatial Durbin model (SDM), one of the most common techniques within the family of spatial data analysis, was employed. Moran’s I statistic was calculated to assess spatial autocorrelation.

The SDM is structured as Y_t_ = pWY_t_ + X_t_β + WX_t_Θ + μ + ε_t_ [18]. The scalar p and K × 1 vector of parameters Θ measure the strength of spatial dependence between units. Y_t_ denotes an Nx1 vector composed of an observation on the outcome variable for every unit in the sample (i = 1, …, N) at time t (t = 1, …, T) and X_t_ denotes an N × K matrix of exogenous explanatory variables at time t associated with the K × 1 vector β. μ indicates an N × 1 vector of ones representing spatial random effects. ε_t_ is an Nx1 vector for independent error terms. The spatial weights matrix W is a positive N × N matrix that describes the structure of interdependence between the sample units [18].

There are few methodological issues that should be mentioned at this stage. First, we applied natural log scale on certain variables, that is, GDP per capita and gasoline amount, in order to obtain better goodness of fit of the models. Second, robust standard error was used. Third, in the SDM, queen contiguity matrix was applied instead of distance weighted matrix. Fourth, all significance tests were assessed at 95% confidence level. Fifth, the results from all models were presented as incidence rate ratio (adjusted IRR) and 95% confidence interval (95% CI). The last point was, in the SDM the incidence of traffic injuries and traffic deaths was transformed into natural log scale first so as to make the regression structured in a log-linear form in compatibility with the other two models. However, the results were exponentiated back a more convenient interpretation during the presentation.

### Assessment of model fitness

We evaluated the accuracy of model prediction by two approaches: (i) data visualisation on Kernel density plots, and (ii) measuring mean absolute error (MAE), mean absolute percentage error (MAPE) and root mean square error (RMSE) for the whole dataset and for each quintile of the dependent variables. Goodness of fit of all models was also measured by Akaike Information Criterion (AIC).

## Results

### Descriptive statistics

From a macro perspective, the incidence of traffic injuries demonstrated a rising trend from 449.0 to 524.9 cases per 100,000 population while traffic deaths fluctuated around 29.7–33.2 deaths per 100,000 population. The case fatality rate showed a slightly decreasing trend, from 0.07 to 0.06 deaths per victim, in the corresponding years, Table 1.

**Table 1:** Incidence proportion of traffic injuries, traffic fatalities and case fatality rate, 2012–2016

Year	2012	2013	2014	2015	2016
All cases—n	298,531	293,880	294,412	321,247	354,073
Deaths—n	21,603	21,221	20,787	19,959	21,745
Population—n × 1000 population	66,492	66,755	67,003	67,236	67,455
Incidence of all cases—n per 100,000 population	449.0	440.2	439.4	477.8	524.9
Incidence of deaths—n per 100,000 population	32.4	31.8	31.0	29.7	32.2
Case fatality rate—deaths per victim	0.07	0.07	0.07	0.06	0.06

**Fig. 1:**
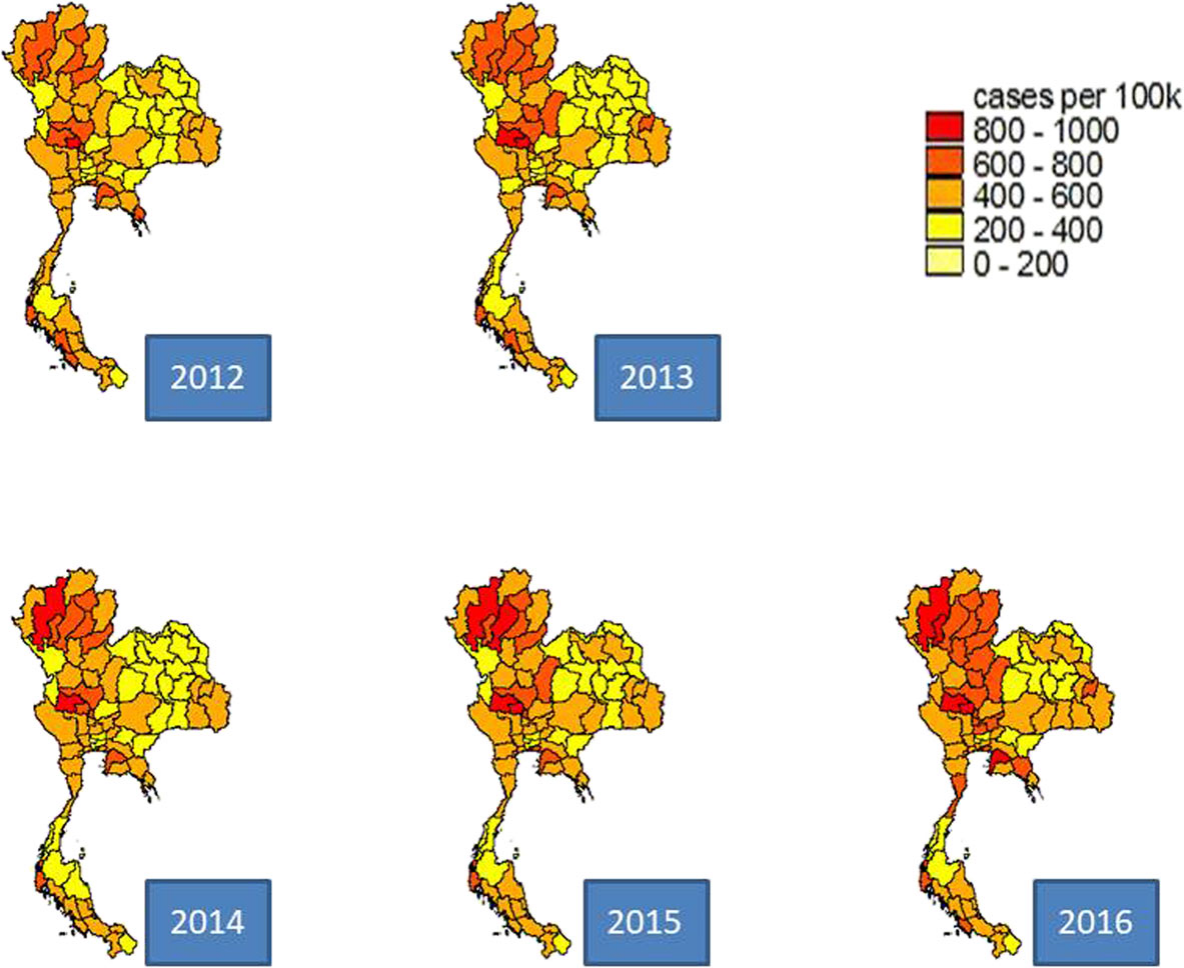
Geographical difference of traffic injuries, 2012–2016

**Fig. 2:**
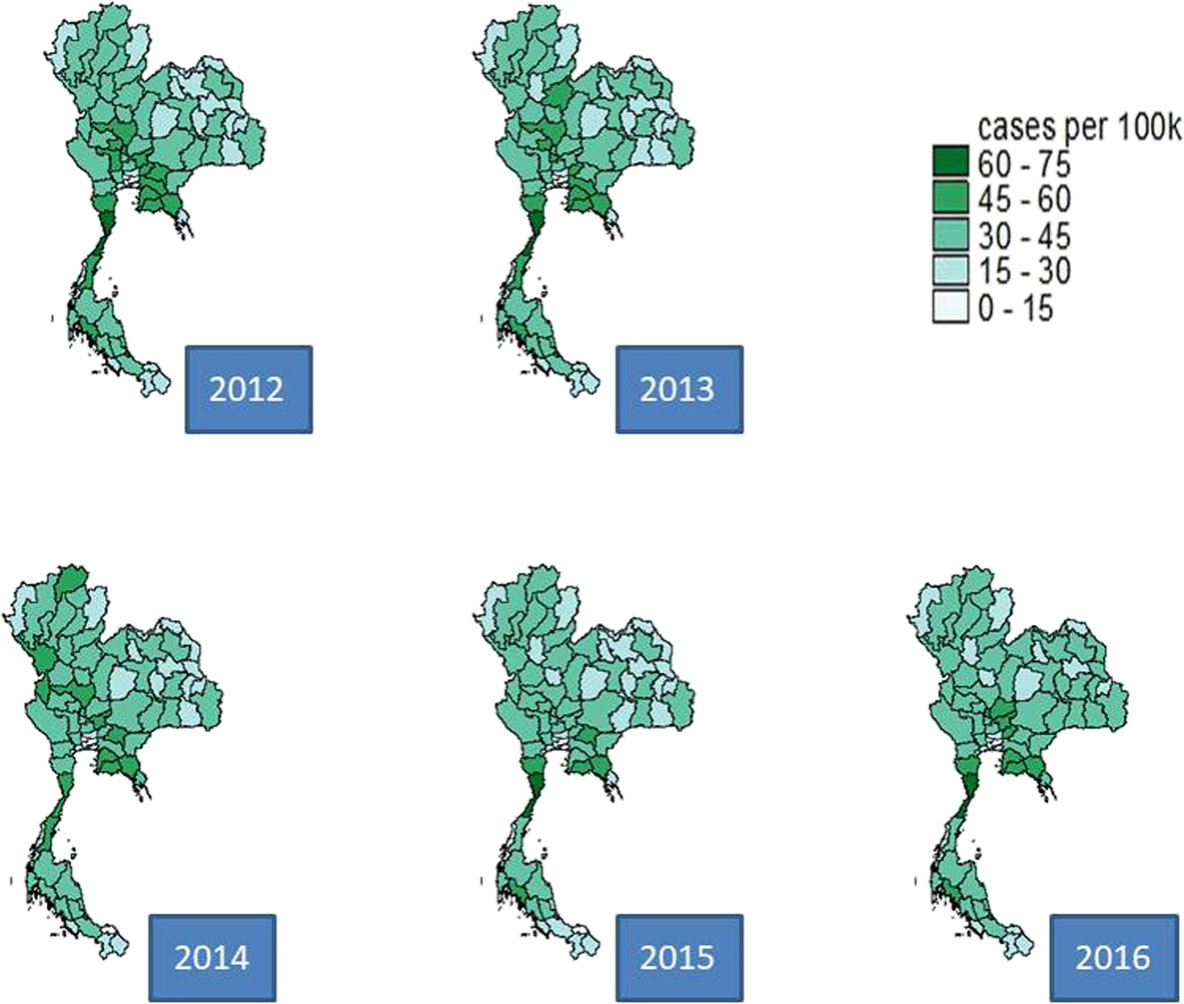
Geographical difference of traffic fatalities, 2012–2016

**Fig. 3:**
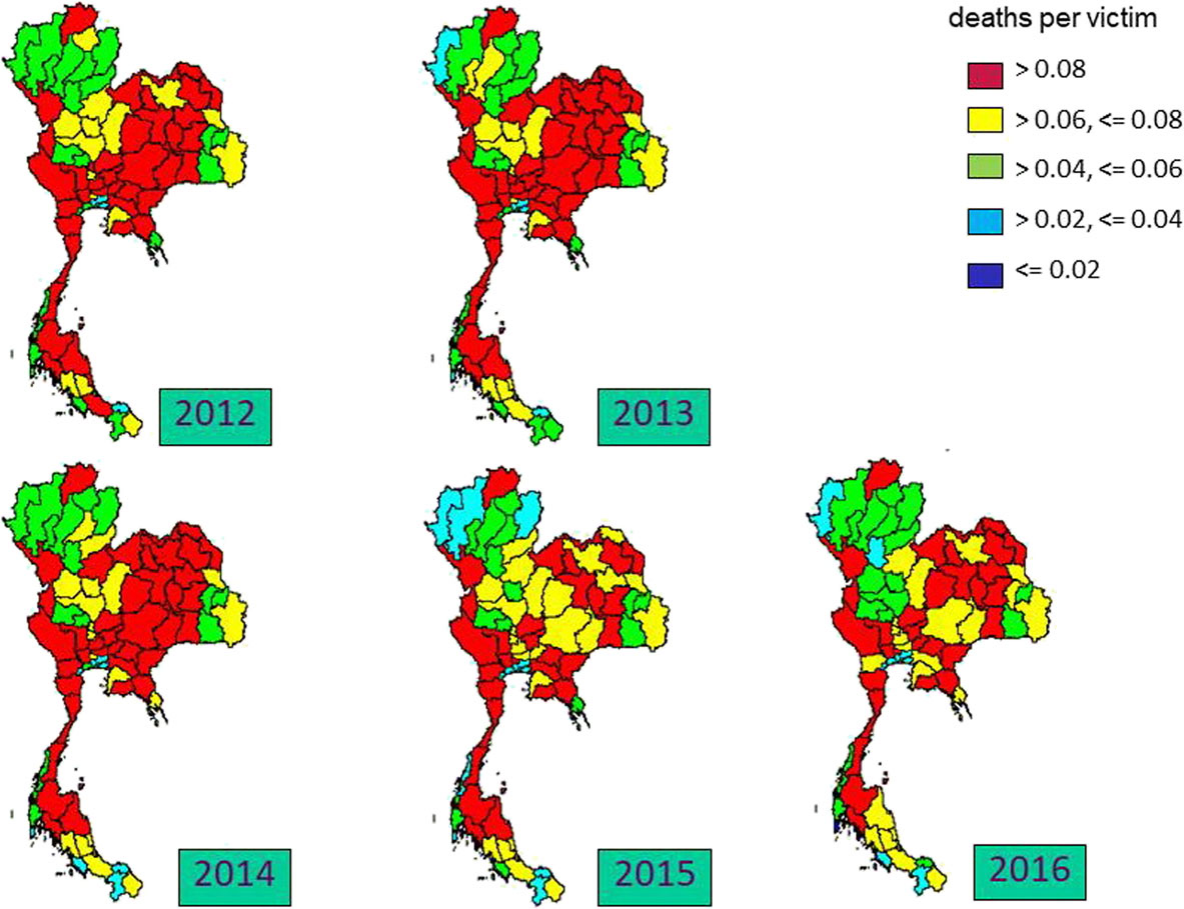
Geographical difference of case fatality rate, 2012–2016

The central and the northern regions saw the largest incidence of traffic injuries. The upper part of the central region encountered the greatest traffic fatality incidence for most years. The geographical difference of traffic deaths was less apparent compared to traffic injuries. Regarding case fatality rate, the country faced a better situation as time passed by. This improvement was most pronounced in the northeastern region, Figs. 1, 2 and 3.

At the provincial level, the mean incidence of traffic injuries varied around 467.3–548.2 cases per 100,000 population whereas the mean incidence of traffic fatalities stood around 32.7–36.0 deaths per 100,000 population. The mean case fatality rate remained stable at 0.07–0.08 deaths per victim. GDP per capita gradually climbed from 143,782.9 Baht (US$ 4357.1) in 2011 to 156,386.8 Baht (US$ 4739.0) in 2016. The fraction of accommodation and restaurant businesses to GDP continuously grew from 1.8 to 2.8%. The manufacturing and industry businesses enlarged alongside the tourism sector, although to a lesser extent (from 25.4 to 25.9%). The amount of gasoline purchased per capita climbed from 383.0 l to 446.2 l during the same period, Table 2.

**Table 2:** Descriptive statistics of traffic injuries, traffic fatalities and all predictor variables across provinces, 2012–2016

Year	2012	2013	2014	2015	2016
Mean incidence of all cases—n per 100,000 population (sd)	486.5 (165.2)	474.8 (166.8)	467.3 (163.5)	507.3 (180.6)	548.2 (185.1)
Median incidence of all cases—n per 100,000 population (iqr)	465.0 (225.0)	463.8 (209.1)	446.2 (199.4)	484.9 (201.5)	528.4 (225.3)
Min/max incidence of all cases—n per 100,000 population	212.7/1033.8	195.1/1055.9	194.1/1067.7	215.3/1265.8	230.3/1364.8
Mean incidence of deaths—n per 100,000 population (sd)	36.0 (11.2)	34.7 (11.0)	33.7 (10.4)	32.7 (10.6)	35.6 (10.2)
Median incidence of deaths —n per 100,000 population (iqr)	35.9 (16.1)	35.4 (17.2)	34.7 (16.0)	32.2 (16.0)	37.3 (13.5)
Min/max incidence of deaths —n per 100,000 population	9.9/61.7	10.1/60.2	9.4/59.6	8.4/61.9	9.8/61.7
Mean case fatality rate—deaths per victim (sd)	0.08 (0.03)	0.08 (0.03)	0.08 (0.04)	0.07 (0.03)	0.07 (0.03)
Median case fatality rate — deaths per victim (iqr)	0.08 (0.05)	0.09 (0.05)	0.08 (0.05)	0.07 (0.04)	0.08 (0.04)
Min/max case fatality rate — deaths per victim	0.03/0.16	0.03/0.16	0.03/0.22	0.02/0.18	0.02/0.16
Mean GDP per capita—Baht (sd)	143,782.9 (140,613.9)	146,311.5 (143,482.1)	146,149.3 (145,653.8)	148,587.9 (145,624.7)	156,386.8 (151,760.4)
Median GDP per capita —Baht (iqr)	99,760.9 (84,423.8)	100,213.1 (84,903.3)	94,414.3 (96,556.7)	92,982.9 (105,215.0)	96,956.9 (104,432.3)
Min/max GDP per capita —Baht	39,376.8/997,061.8	44,641.3/1031.173.0	42,626.2/1031,237.0	45,891.5/986,152.9	49,443.0/1,009,496.0
Mean accommodation and restaurant businesses—% GDP (sd)	1.8 (4.5)	2.1 (5.2)	2.2 (5.2)	2.7 (6.8)	2.8 (7.0)
Median accommodation and restaurant businesses—% GDP (iqr)	0.5 (0.8)	0.6 (1.0)	0.6 (1.1)	0.7 (1.1)	0.7 (1.3)
Min/max accommodation and restaurant businesses—% GDP	0.1/35.6	0.1/38.6	0.1/36.6	0.1/41.2	0.1/43.4
Mean manufacturing and industry businesses—% GDP (sd)	25.4 (18.4)	25.2 (18.1)	25.4 (18.0)	25.6 (17.7)	25.9 (17.4)
Median manufacturing and industry businesses—GDP (iqr)	18.8 (19.2)	18.4 (17.2)	19.8 (18.8)	19.4 (18.1)	20.9 (18.8)
Min/max manufacturing and industry businesses—% GDP	5.6/79.9	6.2/78.3	5.2/77.0	4.9/76.4	4.7/76.0
Mean gasoline purchased per capita—litres (sd)	383.0 (258.1)	393.2 (262.8)	403.2 (288.5)	419.7 (273.1)	446.2 (288.3)
Median gasoline purchased per capita—litres (iqr)	343.5 (292.7)	347.3 (296.3)	331.1 (289.5)	346.9 (309.5)	382.3 (318.7)
Min/max gasoline purchased per capita—litres	67.4/1569.6	72.8/1529.9	79.9/1709.5	102.5/1808.5	121.5/2124.7

*sd* Standard deviation, *iqr* Interquartile range

**Fig. 4:**
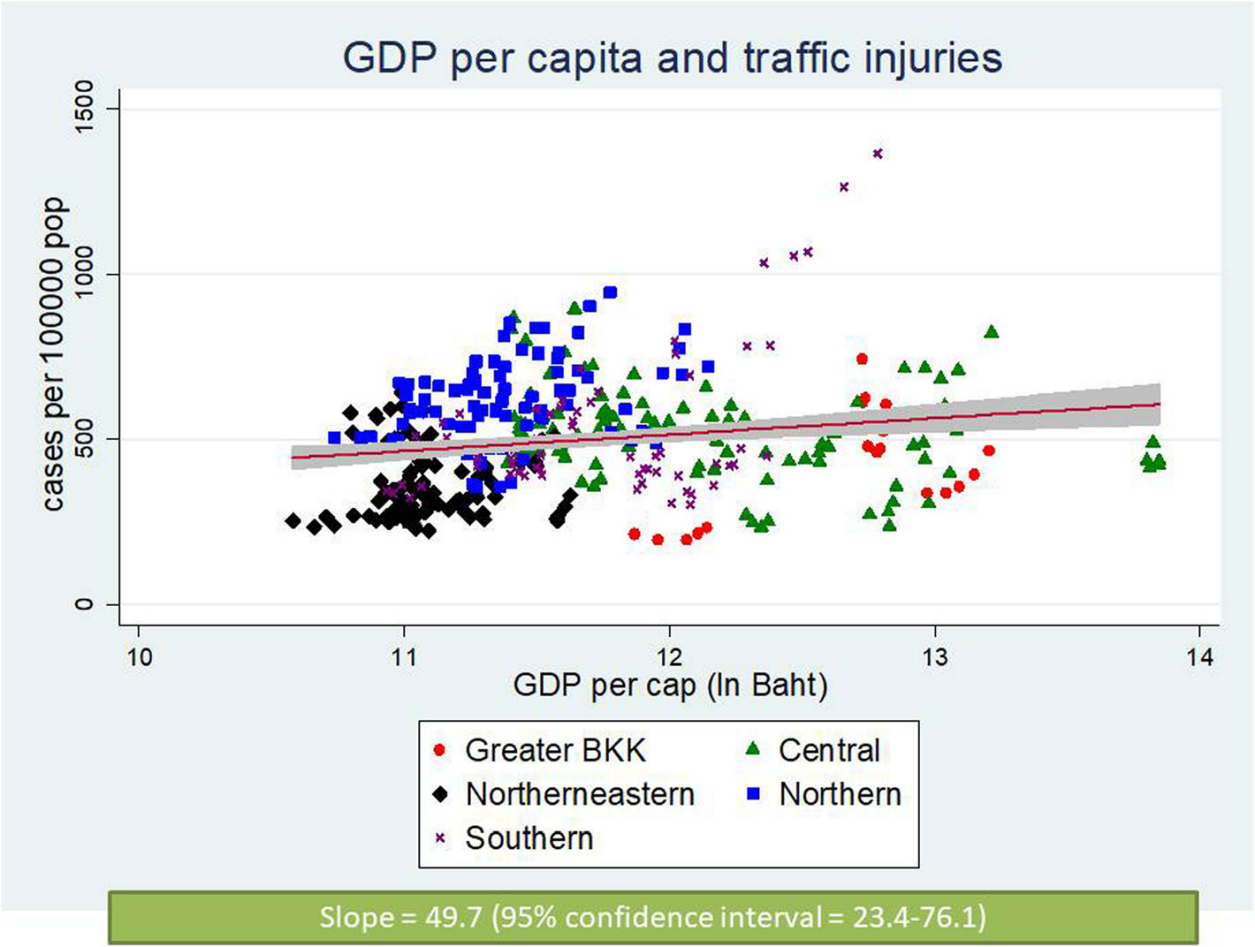
Scatter plot between traffic injuries and GDP per capita, 2012–2016

**Fig. 5:**
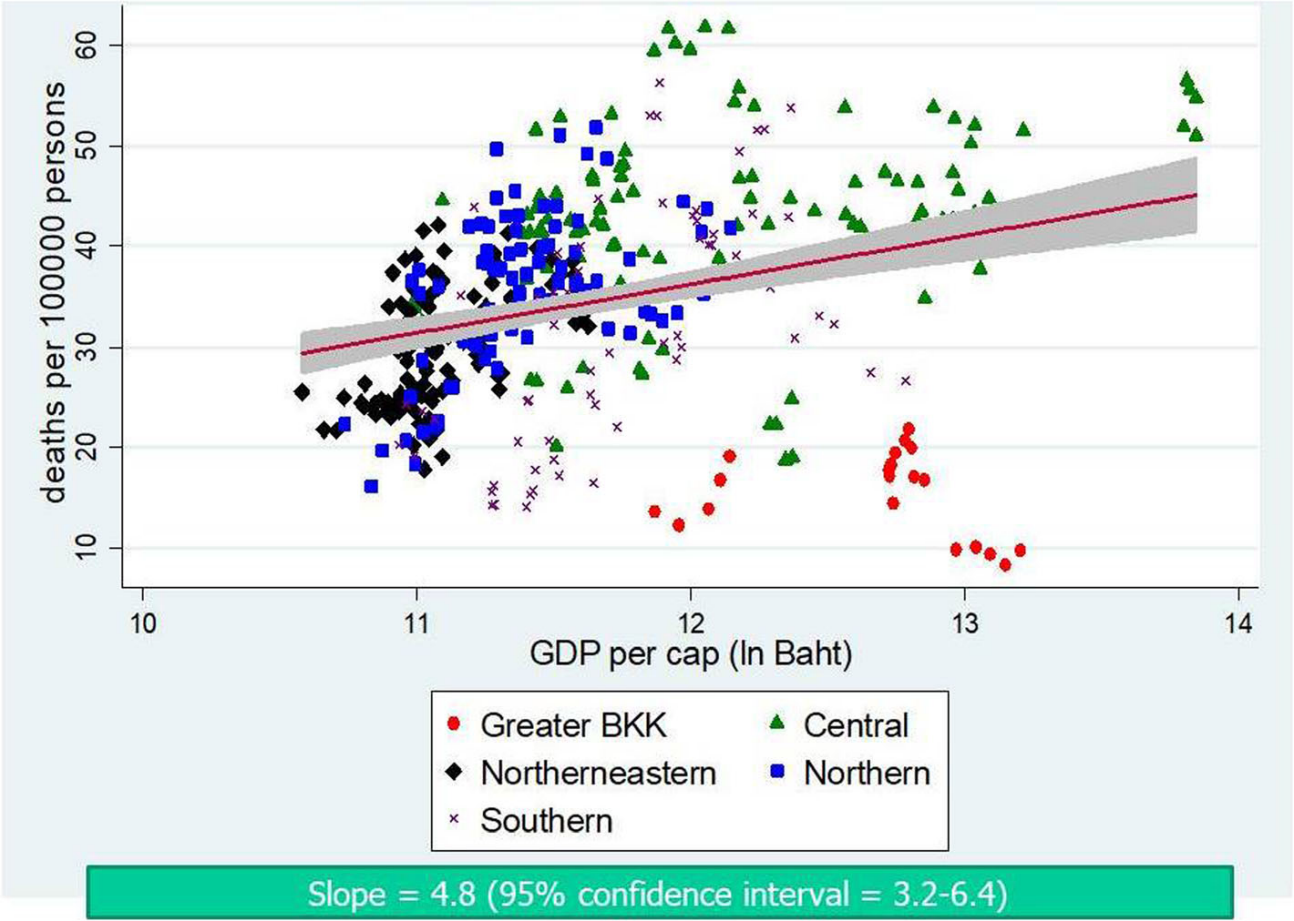
Scatter plot between traffic fatalities and GDP per capita, 2012–2016

**Fig. 6:**
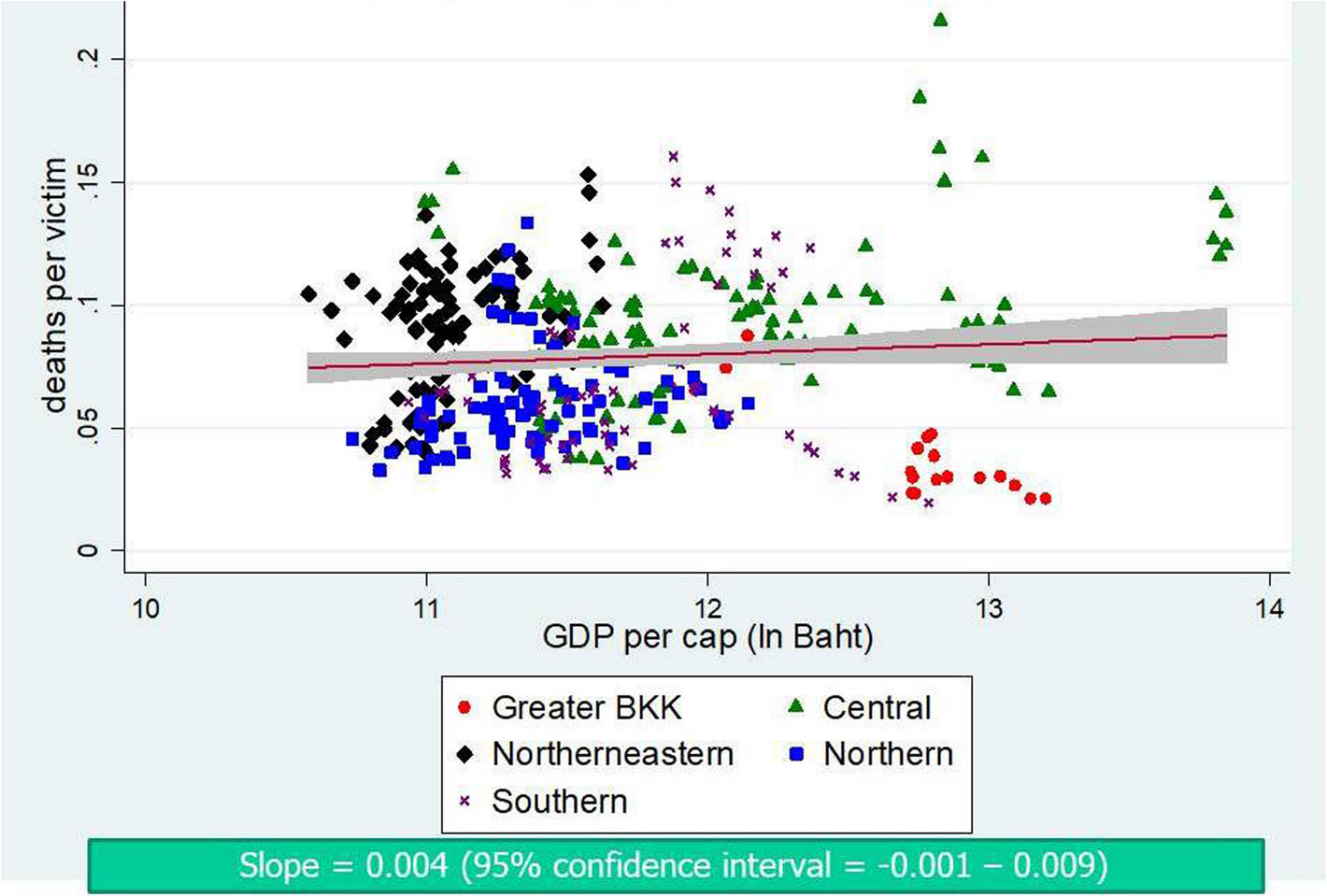
Scatter plot between case fatality rate and GDP per capita, 2012–2016

Figures 4 and 5 contrast the incidence of traffic injuries and traffic deaths against provincial economic level. It is clear that the richer the province; the larger the incidence—confirmed by a positive slope in both graphs. The plots also displayed a clustering pattern of RTIs and GDP per capita by regions. By contrast, as shown in Fig. 6, the fitted line of case fatality rate demonstrated a flatter slope compared to Figs. 4 and 5; and the corresponding 95% CI included zero value—implying a non-significant relationship.

### Negative binomial regression

Table 3 summarises the findings from the NB regression and the RE model. With the NB regression, provincial economy displayed a positive relationship with RTIs incidence notwithstanding no statistical significance found. Being situated in the central region was associated with a 22.3%-increase in injury incidence compared to Greater Bangkok. Also, the incidence was likely to enlarge by 1.7% for a one-percentage increment of the contribution of accommodation and restaurant businesses to provincial GDP per capita. Findings from the RE model showed a similar pattern with a statistical significance found in most variables. In the RE model, a log-Baht increase in GDP per capita significantly expanded injury incidence by around 54.1%; and the whereabouts of the injuries appeared to be a strong determinant.

**Table 3:** Association between outcome variables (traffic injuries, traffic deaths and case fatality rate) and all predictor variables by the NB regression and the RE model

Outcome variables	Predictor variables	Negative binomial regression without random effects			Negative binomial regression with random effects		
		Adjusted IRR	95% CI	*p*-value	Adjusted IRR	95% CI	*p*-value
Traffic injuries	GDP per capita—log Baht	1.033	0.848–1.259	0.749	1.541	1.341–1.771	< 0.001
	Region (reference = Bangkok and its vicinity)						
	• Central	1.223	0.784–1.909	0.374	2.840	2.005–4.025	< 0.001
	• Northeastern	0.942	0.564–1.573	0.819	2.890	1.940–4.305	< 0.001
	• Northern	1.551	0.949–2.533	0.080	4.377	2.992–6.404	< 0.001
	• Southern	1.106	0.675–1.814	0.688	2.951	1.990–4.376	< 0.001
	Gasoline—log litre per capita	1.097	0.926–1.300	0.283	1.075	0.983–1.175	0.112
	Accommodation and restaurant businesses—% GDP	1.017	1.004–1.026	0.008	1.003	0.996–1.010	0.400
	Manufacturing and industry businesses—% GDP	0.997	0.991–1.002	0.258	0.989	0.985–0.993	< 0.001
Traffic deaths	GDP per capita—log Baht	1.087	0.879–1.345	0.440	1.193	1.054–1.351	0.005
	Region (reference = Bangkok and its vicinity)						
	• Central	3.074	2.321–4.071	< 0.001	3.268	2.558–4.175	< 0.001
	• Northeastern	2.726	2.073–3.586	< 0.001	2.803	2.120–3.705	< 0.001
	• Northern	2.994	2.269–3.949	< 0.001	3.138	2.398–4.106	< 0.001
	• Southern	2.527	1.812–3.523	< 0.001	2.629	2.008–3.441	< 0.001
	Gasoline—log litre per capita	1.236	1.097–1.393	0.001	1.048	0.960–1.143	0.297
	Accommodation and restaurant businesses—% GDP	0.997	0.987–1.007	0.577	0.991	0.983–0.999	0.036
	Manufacturing and industry businesses—% GDP	0.998	0.991–1.005	0.599	0.999	0.995–1.003	0.534
Case fatality rate	GDP per capita—log Baht	1.006	0.752–1.345	0.969	0.827	0.704–0.968	0.019
	Region (reference = Bangkok and its vicinity)						
	• Central	2.251	1.380–3.670	0.001	2.599	1.783–3.787	< 0.001
	• Northeastern	2.544	1.455–4.449	0.001	2.062	1.356–3.137	0.001
	• Northern	1.678	0.977–2.881	0.060	1.534	1.018–2.313	0.041
	• Southern	2.047	1.157–3.620	0.014	2.066	1.371–3.112	0.001
	Gasoline—log litre per capita	1.160	0.914–1.471	0.221	0.852	0.762–0.953	0.005
	Accommodation and restaurant businesses—% GDP	0.985	0.965–1.005	0.145	0.985	0.975–0.996	0.009
	Manufacturing and industry businesses—% GDP	1.002	0.995–1.009	0.004	1.009	1.003–1.014	0.001

*95% CI* 95% confidence interval, *IRR* Incidence rate ratio

In the angle of traffic fatalities, provincial economy did not display a significant association by the NB regression. The upcountry saw a larger incidence by around 2.5–3.1 times compared to Greater Bangkok. Every one log-Baht increase in GDP per capita resulted in the growth of traffic fatalities by approximately 8.7%. This figure was greater in the RE model, amounting to 19.3%. The adjusted IRR of other covariates in both models was close to one another.

Concerning case fatality rate, GDP per capita demonstrated a more-than-one adjusted IRR in the NB regression yet without statistical significance. On the contrary, the NB regression exhibited a reverse relationship, denoting that a log-Baht increase in GDP per capita would result in a 17.3% decrease in case fatality rate. Accidents outside Bangkok seemed to face greater case fatality rate than inside Bangkok.

### Spatial panel data analysis

The SDM showed that for a log-Baht increase in GDP per capita, the incidence of traffic injuries would enlarge by approximately 30.7%; while in traffic fatalities, a 23.8%-increase of the incidence was observed. Statistical significance was noted in both outcome variables. All regions but the South tended to face larger incidence of traffic deaths compared to Greater Bangkok. No statistical significance was found in the analysis of GDP per capita on case fatality rate. Global Moran’s I exhibited a statistical significance with *p*-value smaller than 0.001 in almost all years of interest. This warranted the use of the SDM to account for spatial autocorrelation of the data, Table 4.

**Table 4:** Association between outcome variables (traffic injuries, traffic deaths and case fatality rate) and all predictor variables by the SDM

Outcome variables	Predictor variables	Adjusted IRR	95% CI	*P*-value
Traffic injuries	GDP per capita—log Baht	1.307	1.147–1.489	< 0.001
	Region (reference = Bangkok and its vicinity)			
	• Central	1.341	0.830–2.166	0.230
	• Northeastern	1.564	0.832–2.937	0.165
	• Northern	1.907	0.970–3.749	0.061
	• Southern	0.785	0.439–1.404	0.414
	Gasoline—log litre per capita	1.045	0.960–1.139	0.308
	Accommodation and restaurant businesses—% GDP	0.997	0.992–1.002	0.196
	Manufacturing and industry businesses—% GDP	0.994	0.990–0.998	0.002
Traffic deaths	GDP per capita—log Baht	1.238	1.044–1.469	0.014
	Region (reference = Bangkok and its vicinity)			
	• Central	2.164	1.557–3.008	< 0.001
	• Northeastern	1.887	1.246–2.857	0.003
	• Northern	2.198	1.499–3.225	< 0.001
	• Southern	1.667	0.573–4.847	0.348
	Gasoline—log litre per capita	1.090	1.001–1.187	0.047
	Accommodation and restaurant businesses—% GDP	0.996	0.988–1.004	0.345
	Manufacturing and industry businesses—% GDP	0.999	0.994–1.004	0.678
Case fatality rate	GDP per capita—log Baht	0.971	0.789–1.194	0.779
	Region (reference = Bangkok and its vicinity)			
	• Central	1.550	0.991–2.423	0.055
	• Northeastern	1.223	0.732–2.044	0.443
	• Northern	1.141	0.622–2.095	0.430
	• Southern	1.983	0.393–10.012	0.407
	Gasoline—log litre per capita	1.000	0.876–1.142	0.999
	Accommodation and restaurant businesses—% GDP	0.997	0.992–1.003	0.331
	Manufacturing and industry businesses—% GDP	1.006	1.001–1.012	0.027

The Global Moran’s I statistics between 2012 and 2016 equated 0.394 (*p*-value < 0.001), 0.383 (*p*-value < 0.001), 0.385 (*p*-value < 0.001), 0.336 (*p*-value < 0.001) and 0.303 (*p*-value < 0.001) in traffic injuries; equated 0.333 (*p*-value < 0.001), 0.286 (*p*-value < 0.001), 0.256 (*p*-value < 0.001), 0.259 (*p*-value < 0.001) and 0.289 (*p*-value < 0.001) in traffic deaths; and equated 0.300 (*p*-value < 0.001), 0.313 (*p*-value < 0.001), 0.222 (*p*-value = 0.001), 0.222 (*p*-value = 0.001), and 0.288 (*p*-value < 0.001) in case fatality rate; *95% CI* 95% confidence interval, *IRR* Incidence rate ratio

**Fig. 7:**
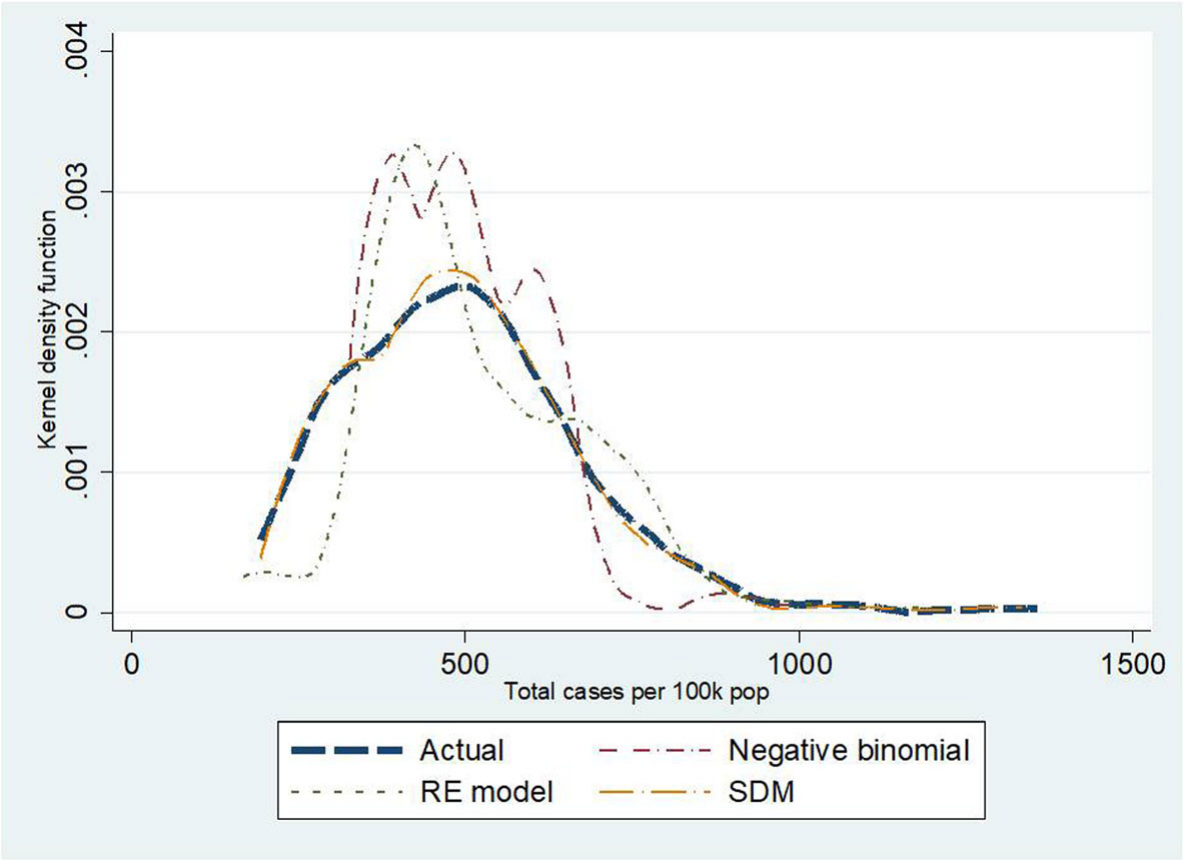
Kernel density plots for traffic injuries by different models

**Fig. 8:**
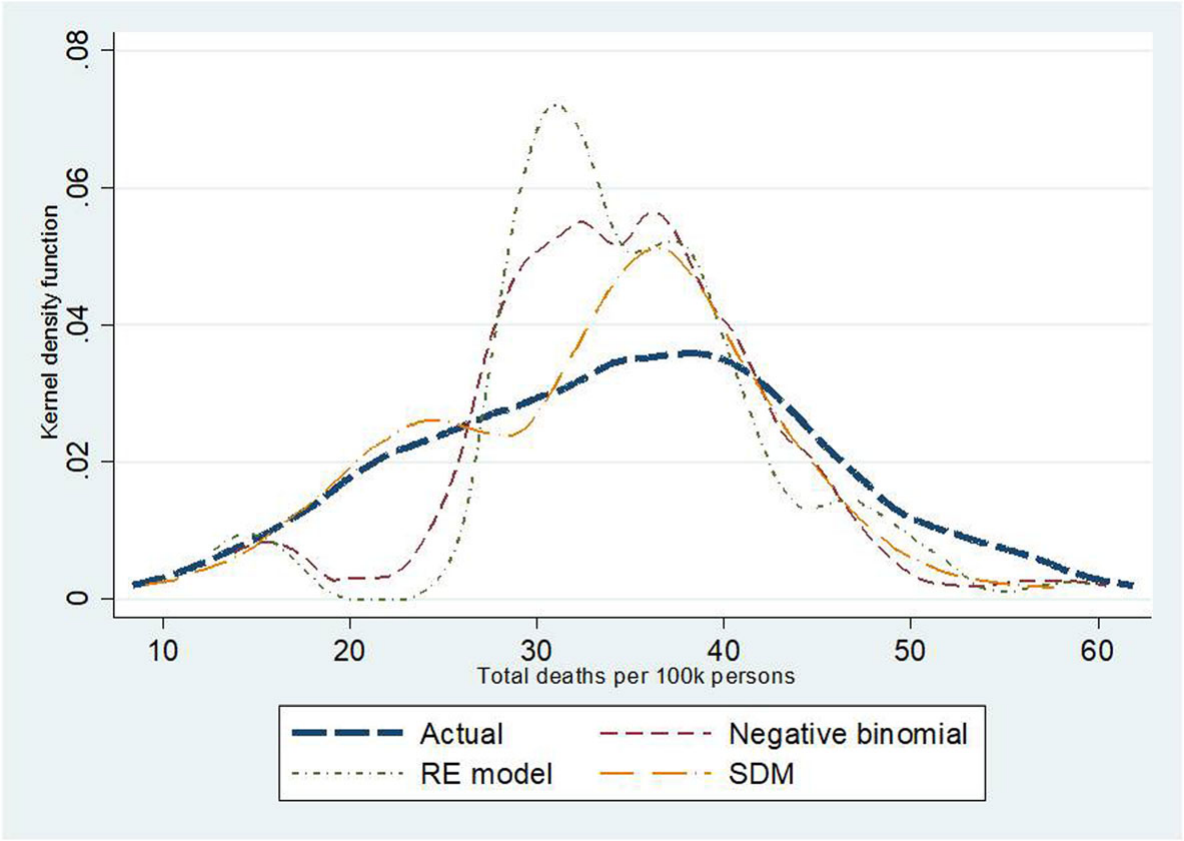
Kernel density plots for traffic fatalities by different models

**Fig. 9:**
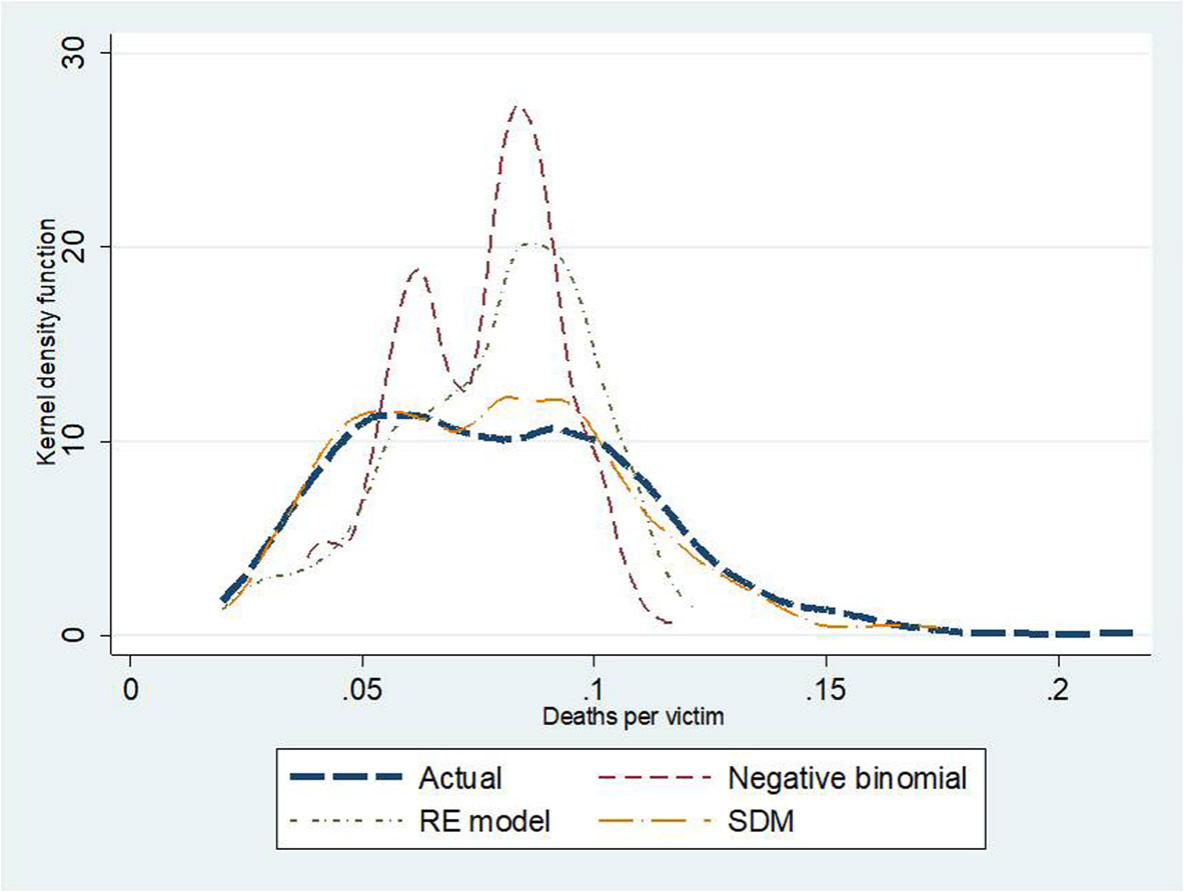
Kernel density plots for case fatality rate by different models

Kernel density plots indicate that the SDM seemed to be the best fitted model amongst the three candidate models. This observation was confirmed by the smallest values of MAE, MAPE and RMSE in the SDM relative to the other models. For instance, in traffic deaths, MAPE in the NB regression and the RE model amounted to 19.6–20.9; about twice the size of MAPE in the SDM. When exploring quintile by quintile, the second and the third quintiles showed the best predictive power compared to the other quintiles. Also, the SDM saw the lowest AIC—suggesting more favourable model fitness over the other models. More details on this point are presented in Tables 5 and 6 and Figs. 7, 8 and 9.

**Table 5:** MAE and MAPE in different models for the whole dataset and for each quintile of the outcome variables

Data	Models	All cases			Deaths			Case fatality rate		
		MAE	MAPE	RMSE	MAE	MAPE	RMSE	MAE	MAPE	RMSE
All data	Negative binomial regression without random effects	99.5	22.6	127.3	5.8	19.6	7.5	0.02	28.6	0.03
	Negative binomial regression with random effects	112.1	24.9	150.4	6.2	20.9	7.9	0.02	31.6	0.03
	SDM	25.3	5.3	33.0	3.9	8.6	4.0	0.01	8.6	0.01
1st quintile	Negative binomial regression without random effects	118,1	44.7	139.8	4.3	18.4	6.5	0.02	58.2	0.03
	Negative binomial regression with random effects	106.7	38.5	128.3	4.8	20.6	7.1	0.02	62.4	0.03
	SDM	16.9	6.1	20.5	2.2	7.7	2.8	< 0.01	9.9	< 0.01
2nd quintile	Negative binomial regression without random effects	81.9	21.2	109.4	5.9	20.5	7.6	0.01	22.2	0.02
	Negative binomial regression with random effects	113.9	29.1	162.9	6.9	23.0	8.4	0.02	32.0	0.02
	SDM	21.1	5.4	26.9	3.0	7.8	3.8	< 0.01	8.2	0.01
3rd quintile	Negative binomial regression without random effects	70.2	14.5	87.0	6.6	24.8	7.8	0.01	17.9	0.02
	Negative binomial regression with random effects	95.3	19.8	131.9	7.0	26.7	8.4	0.02	19.9	0.02
	SDM	23.5	4.9	31.0	3.1	8.7	4.2	0.01	7.8	0.01
4th quintile	Negative binomial regression without random effects	82.0	14.3	103.9	6.6	19.9	8.4	0.01	13.4	0.02
	Negative binomial regression with random effects	117.9	20.6	150.5	7.1	21.2	8.7	.0.01	12.2	0.02
	SDM	29.7	5.2	38.4	3.1	9.1	4.6	0.01	8.1	0.01
5th quintile	Negative binomial regression without random effects	145.5	18.5	176.7	5.4	14.5	7.2	0.04	31.5	0.05
	Negative binomial regression with random effects	126.7	16.8	173.3	4.9	12.9	6.6	0.04	31.4	0.05
	SDM	35.4	4.8	43.0	3.6	9.5	4.2	0.01	8.9	0.01

*SDM* Spatial Durbin model, *MAE* Mean absolute error, *MAPE* Mean absolute percentage error, *RMSE* Root mean square error

**Table 6:** Goodness of fit of the models as measured by AIC

Model	Traffic injuries	Traffic deaths	Case fatality rate
Negative binomial regression without random effects	6284.1	4126.4	4411.9
Negative binomial regression with random effects	5804.8	3771.8	3940.3
SDM	−671.8	− 379.1	−269.1

## Discussion

Overall, traffic injuries presented a positive correlation with provincial prosperity. This relationship remained after the key covariates like geographical idiosyncratic features (region), traffic intensity (gasoline purchased per capita), touristy (accommodation and restaurant businesses) and industrial activities (manufacturing and industry businesses) were adjusted. The analysis on traffic deaths came up with the same direction.

For every log-Baht increase in GDP per capita (which was equivalent to a growth of GDP per capita by about 2.7 times), the incidence of traffic injuries and traffic deaths would significantly expand by approximately 23.8–30.7% if the SDM was applied with all else being equal. This discovery alludes to the fact that the effectiveness of pre-crash management in a province might not go in tandem with provincial economic growth. Interestingly, provincial prosperity yielded a reverse relationship with case fatality rate despite no statistical significance coming out. One of the likely explanations for this discovery is the instigation of policy that promotes equitable post-crash management throughout the country (independent of provincial prosperity). A concrete instance for this presumption is the Universal Coverage for Emergency Patients (UCEP) policy launched in 2017 [19]. The essence of the UCEP is all public and private hospitals are obliged to treat emergency patients with free of charge for the first 72 h. Such a measure partly alleviates barriers in healthcare access and patient referral systems that are due to financing reasons. Besides, the post-crash services (including ambulances, life-rescuing equipment and trained personnel) are now in place in all districts; starting from the elementary unit of care, that is, district hospitals. Patients suffering from severe injuries can be referred to higher-level facilities (for example, regional and university hospitals), mostly located in economically well-off provinces, with no cost. These measures were implemented alongside a more stringent supervision on emergency care standards and referral system by the National Institute for Emergency Medicine. All of these accounts helped illuminate why case fatality rate did not show a significant difference across regions despite a slight favourable outcome found in provinces with better economic status. Yet this conjecture still lacks empirical evidence and warrants much further research.

Another influential determinant of RTIs and traffic deaths is region-specific characteristic. Larger incidence of traffic fatalities seemed to occur outside Greater Bangkok. Some likely explanations are that the upcountry usually face less-safe road conditions and poorer law enforcement compared to Greater Bangkok. Besides, in particular areas, like the northern region, there are numerous mountains and highlands. These geographical features may partly contribute to RTIs. This supposition coincides with the study by Berecki-Gisolf et al., which found that the northern region of Thailand was more likely to experience car crashes relative to other regions [20].

To our knowledge, this piece of work is amongst the first few studies that seek to determine the implication of subnational economy on traffic injuries/deaths in Southeast Asia. Suriyawongpaisal and Kanchanasut had ever explored similar issue before 2000, but at that time only descriptive statistics were used. The authors also suggested that traffic-injury trend seemed to follow economic growth and summarised that the number and rate of traffic injuries in Thailand swung markedly during the 1980s, reached the record high during the bubble economy era in early 1990s, then subsided during the Asian economic crisis in 1997 [21].

The positive influence of subnational economy on RTIs may not be due to the growing intensity of vehicle used and the increased human mobility alone (since we had already accounted for those factors in the model) and there may be some other explanations for this phenomenon. Iwata explained that in economically active areas people’s marginal utilities for using vehicles are larger than those for protecting traffic accidents [22]. Bishai et al. explored RTIs data from 41 countries (excluding Thailand) between 1992 and 1996 and discovered that a 10% rise in GDP in a low-income country was expected to accrue the number of traffic injuries by 4.7%, and the number of deaths by 3.1% through a mechanism that is independent of population size, vehicle counts, gasoline use, and roadway availability [23]. By contrast, increases in GDP in developed countries appeared to reduce the death tolls from traffic accidents. Bishai et al. concluded that the rise-and-fall pattern when plotting traffic fatalities against economic growth aligned with ‘Kuznets inverted-U curve theory’ [23]. The curve is widely utilised in environmental research and is explained as, in an early stage of development, the centre of a nation’s economy shifts towards industrialisation, and this tends to accelerate environmental degradation until average income reaches a certain point over the course of development [24]. At a certain threshold, economic centre will shift from industrial businesses to service-sector businesses and investments in harm reduction will increase to keep pace with the growth of pollution producing activities [25].

By considering vehicle crashes as harm in the same way as exhaust fumes, some health economists and/or epidemiologists used the curve to identify the relationship between RTIs and economic development [23]. Van Beeck et al. corroborated this idea by plotting accident mortality against country prosperity in 21 industrialised countries from 1962 till 1990. They found a positive relation between nation prosperity and traffic mortality during the 1960s, but at a certain economic point (GDP per capita = US$ 3000) the mortality rate decelerated [26]. One of key challenges to find a solid conclusion based on this concept is different studies suggest different turning point. Bishai et al. concluded that the threshold for traffic fatality rate to decline varied around US$ 1500 – US$ 8600 [23]. Kopits and Cropper pointed to the threshold at US$ 8600 [27] while Anbarci et al. indicated a slightly higher bar, at US$ 11,454 [16]. Note that the comparison between these thresholds should be made with caution as they were analysed by different statistical models and in diverse settings and timeframes.

There are several explanations for this up-and-down pattern, such as a ‘competing risks story’ in which it is sensible for motorists in LMICs to underinvest in road safety and bear a greater exposure to high risk transport options in order to generate more income which can be used to control other health problems (for instance, infectious diseases and malnutrition). Conversely, in HICs, those health problems recede in importance relative to transport injuries; hence it becomes rational for motorists to invest more resources on road safety [23]. Another explanation is a ‘vehicle mix story’ in which lower traffic fatality rates will come out when the level of economy reaches certain point. This is because economic growth empowers more road users to switch from dangerous transport modalities (such as motorcycles or rooftops on buses) to safer modalities (such as sedan cars and trains).

Of note is that our analysis used subnational data while the aforementioned literature used national-level data. Therefore comparing our findings with the theory is not always straightforward. However, if the theory holds true in the backdrops of the Thai RTIs situation, it is worth questioning whether Thailand has reached the turning point on the inverted-U curve—if not, where the threshold is and when to reach it (as of 2017, the Thai GDP per capita = US$ 6593.8 [28]). With respect to our findings, Thailand is still on an upward trend on the Kuznets curve as supported by a rising slope in scatter plots (Figs. 4 and 5) and a more-than-one IRR from the analysis on traffic injuries and traffic deaths in all models (Tables 3 and 4).

In terms of policy implications, we by no means intend to flag that the Thai Government should wait until a certain level of prosperity then RTIs will subside spontaneously. By contrast, all concerted efforts to prevent RTIs and care for RTIs victims should continue and even be more intensified. Should we believe that RTIs are (partly) attributed by provincial economy and, undeniably, the prosperity of a province is a result of the investment of large entrepreneurs from outside that province, it is imperative to question whether and to what extent the entrepreneurs and business sectors should share responsibility in RTIs prevention in the areas where they are operating. Recent study by Nithichai pointed that the average annual growth rate of residents’ income in provinces having a great number of giant retail stores (whose business owners were in Greater Bangkok) was as high as 5.6%; and during 2006–2011 the total number of these giant retail stores in the upcountry enlarged by 85.4% [29]. This evidence in combination with our findings ratifies the notion of ‘Corporate social responsibility’ (CSR), the widely accepted concept for nowadays economy. To be more specific, there should be a legal mechanism that requires the entrepreneurs and the business sectors to invest in RTIs prevention in areas where theirs businesses are running. However this proposal necessitates much more investigation on the political feasibility and implementation details.

Methodology-wise, this study bears some strengths and weaknesses. Concerning strength, our study has employed statistical technique, which simultaneously captures influence of space and time. We attest that in spatially-correlated data like RTIs, the application of spatial panel data analysis is more justified than conventional statistical models.

Yet there remain some limitations and/or points of concern. Firstly, traffic-injury and death data were obtained from different sources and death data were not a subset of injury data. To be more specific, the injury data were retrieved from the E-claim—which compiled events with claim petitions only. As a result, any non-claim events, for instance, minor bumpings or crashes in non-registered vehicles, were likely to be excluded. Nevertheless this point might not lead to any serious bias as we intended to identify the relationship between RTIs (plus traffic deaths and case fatality rate) and subnational economy rather than determining the total magnitude of the outcome variables. As briefly mentioned in the Methods section, we had explored other alternative data sources, particularly, the POLIS and the IS, but finally we decided to use the E-claim as it contained the larger volume of data than the other two data sources. The POLIS gathered only vehicle crashes that were investigated by the police or state prosecutors. The IS only had information of RTIs victims who visited MOPH-affiliated health facilities. As a result, the IS lacked data of injury and death cases that did not show up at public health facilities. While the system for collecting and merging death data from all three sources (E-claim, IS and Death Registry) is now being in function by the NCD-DDC, the data-collecting system for injury cases is yet to be developed. This can be viewed as room for improvement for both policy makers and relevant health-science academics.

Secondly, like in all studies, the concern over data quality is of importance. It is always difficult to assess if the data collected really reflected what happened in the field. However this point might not be that critical in the E-claim and the NCD-DDC datasets. This is because the E-claim is routinely used for claim petition and reimbursement. As a result, the data submission should be detailed as much as possible to avoid the indemnity loss. For death records, we also checked the cleanliness of the data (which were basically cleaned by the NCD-DDC in the first instance) by examining all outliers and data patterns before undertaking the analyses.

Thirdly, many variables included in the models were a surrogate measurement, for instance gasoline used as a proxy for traffic intensity. This means the audiences should be heedful when comparing our findings with other studies that apply different measurements.

Last but not least, there are other unobserved variables that might affect the analysis but had not been enrolled in the model. These include, but are not limited to, distance of road networks, level of education of motorists, road safety investment, urbanisation, legislation and compliance with traffic laws amongst the motorists. We attempted to explore these variables but unfortunately not all of them were not publicly available. In addition, it is very difficult to quantify these variables, except for urbanisation which can be in part captured by GDP per capita and percentage contribution of manufacturing businesses. In 2016 the Thailand National Status Report on Road Safety used to assess the effectiveness of RTIs-prevention performances in all provinces in Thailand. However, it was still a subjective (self) assessment through the lens officers in the field and the like assessment before 2016 was lacking. Future research that takes into account these determinants are definitely of great value.

## Conclusion

The incidence of traffic injuries and traffic deaths demonstrated a positive correlation with provincial prosperity after adjusting for potential covariates including traffic intensity and economic contribution of touristy, manufacturing and industry businesses. For a log-Baht increment in GDP per capita (or about a 2.7-fold growth in provincial economy), traffic injuries and deaths were likely to increase by about a quarter. Nevertheless, no significant relationship between provincial economy and case fatality rate was found. Methodology-wise, the SDM seemed to be the best fitted model compared to the NB regression and the RE model. Larger incidence of traffic fatalities appeared to occur outside Greater Bangkok. Future research that captures unobserved but important RTIs determinants, such as distance of road networks, education profile of provincial residents, public investment in transportation safety, and compliance with traffic laws amongst the motorists, is recommended.

## Data Availability

The datasets used and/or analysed during the current study are available from the corresponding author on reasonable request.

## Abbreviations

95% CI: 95% confidence interval
AIC: Akaike information criterion
CCS: Country Cooperation Strategy
CSR: Corporate social responsibility
DEB: Department of Energy Business
FE: Fixed effect
GDP: Gross domestic product
HICs: High-income countries
IRR: Incidence rate ratio
IS: Injury Surveillance System
LMICs: Low- and middle-income countries
MAE: Mean absolute error
MAPE: Mean absolute percentage error
MOPH: Ministry of Public Health
NB: Negative binomial
NCD-DDC: The Division of Non-Communicable Diseases of the Department of Disease Control (NCD-DDC)
NESDB: Office of the National Economic and Social Development Board
POLIS: Royal Thai Police Information System
RE: Random effects
RMSE: Root mean square error
RTIs: Road traffic injuries
RVP: Road Accident Victims Protection Company
UCEP: Universal Coverage for Emergency Patients
WHO: World Health Organization
